# Replicating associative learning of rodents with a neuromorphic robot in an open-field arena

**DOI:** 10.3389/fnins.2025.1565780

**Published:** 2025-06-25

**Authors:** Tianze Liu, Kang Jun Bai, Hongyu An

**Affiliations:** ^1^Department of Electrical and Electronic Engineering, Michigan Tech, Houghton, MI, United States; ^2^Air Force Research Laboratory, Rome, GA, United States; ^3^Department of Electrical and Electronic Engineering, Michigan Tech, Houghton, MI, United States

**Keywords:** associative learning, Hebbian learning, neuromorphic computing, robot, SNN algorithm

## Abstract

This study emulates associative learning in rodents by using a neuromorphic robot navigating an open-field arena. The goal is to investigate how biologically inspired neural models can reproduce animal-like learning behaviors in real-world robotic systems. We constructed a neuromorphic robot by deploying computational models of spatial and sensory neurons onto a mobile platform. Different coding schemes—rate coding for vibration signals and population coding for visual signals—were implemented. The associative learning model employs 19 spiking neurons and follows Hebbian plasticity principles to associate visual cues with favorable or unfavorable locations. Our robot successfully replicated classical rodent associative learning behavior by memorizing causal relationships between environmental cues and spatial outcomes. The robot’s self-learning capability emerged from repeated exposure and synaptic weight adaptation, without the need for labeled training data. Experiments confirmed functional learning behavior across multiple trials. This work provides a novel embodied platform for memory and learning research beyond traditional animal models. By embedding biologically inspired learning mechanisms into a real robot, we demonstrate how spatial memory can be formed and expressed through sensorimotor interactions. The model’s compact structure (19 neurons) illustrates a minimal yet functional learning network, and the study outlines principles for synaptic weight and threshold design, guiding future development of more complex neuromorphic systems.

## 1 Introduction

In recent years, deep learning and artificial intelligence (AI) have achieved remarkable advancements, especially in pattern recognition, classification, computer vision, and natural language processing (Voulodimos et al., 2018, Ismail Fawaz et al., 2019, Chowdhary and Chowdhary, 2020, Yang et al., 2020, Bai et al., 2021, Eskandari et al., 2021). The remarkable capabilities of these deep learning approaches stem from rigorous training processes involving extensive datasets and large-scale Artificial Neural Networks (ANNs). During training, ANNs compare their outputs against labeled ground truth data, and errors are backpropagated through the entire neural network. This process minimizes error via the loss function, achieved by iterative weight adjustments using optimization algorithms. Thus, larger datasets and more complex neural networks generally lead to higher accuracy (Goodfellow et al., 2016a,Devlin et al., 2018), resulting in a persistent demand for ever-expanding datasets and network scales (Goodfellow et al., 2016a; Sun et al., 2017; Devlin et al., 2018). However, this dependence on large-scale data and labeled data samples introduces critical challenges, including high power consumption, data scarcity, and reduced flexibility in autonomous operations. These challenges render ANNs unsuitable for applications with strict Size, Weight, and Power (SWaP) constraints from the on-field robots (Goodfellow et al., 2016b,Devlin et al., 2018). For instance, planetary rovers need to possess high adjustability and autonomous operating capabilities with minimal human intervention in environments characterized by constrained energy sources and communications (Devlin et al., 2018).

To overcome these challenges, we enhance the autonomous operating capabilities of intelligent robots by emulating the associative learning of rodents using neuromorphic computing and robots. Associative learning is a pervasive self-learning mechanism observed across diverse animal species. Associative learning presents the ability to adapt to the environment by interacting with their surroundings and memorizing concurrent events (Kandel et al., 2000, Kohonen, 2012, Sun et al., 2019). The exploration and learning of rodents in an open-field arena demonstrate classic associative learning behavior. In an open-field arena, rodents are exposed to distinct stimuli or cues, which may include visual, auditory, or a combination of sensory inputs. These stimuli are categorized into conditional and unconditional stimuli. For example, in the classic Barnes maze (Barnes, 1988, Barnes et al., 2005), the escape hole is considered the unconditional stimulus, while the neutral visual cues are treated as conditional stimuli. Through repeated exposure, the rodents gradually discern the predictive relationships between the presented cues and their corresponding outcomes. One specific outcome is a direct movement trajectory toward the escape hole guided by the visual cues. The associative learning has significant potential to enhance robots by enabling them with self-learning and exploration. In dynamic and unknown environments, such as on the Moon and Mars, planetary rovers equipped with associative learning capabilities can explore unknown areas and autonomously adapt their behavior. Several studies have implemented associative learning at the simulation level (Eryilmaz et al., 2014, Moon et al., 2014, Moon et al., 2014, Hu et al., 2015, Liu et al., 2016, Hu et al., 2017, Yang et al., 2017, An et al., 2019, Sun et al., 2019). However, these investigations face several limitations, including the use of small-scale neural networks, a reliance on purely simulation-based approaches rather than experimental validations, and the lack of deployment on robotic platforms to test real-world scenarios (Eryilmaz et al., 2014, Moon et al., 2014, Liu et al., 2016, Hu et al., 2017, Yang et al., 2017).

This paper presents a novel associative learning model that enables a real-time self-learning capability. The self-learning capability is validated by replicating the spatial learning tasks of rodents in a circular and open-field arena without pretraining and labeled datasets. Our associative learning model leverages Hebbian principles (Kandel et al., 2000, Sosa and Giocomo, 2021; Zins et al., 2023a; Zins et al., 2023b; Liu et al., 2024), specifically Oja’s rule, to dynamically adjust synaptic weights and build associations between sensory neurons and response neurons in real-time. Our neuromorphic robot system demonstrates adaptive behavior by memorizing visual cues, such as red color markers, with vibration signals. In addition to Hebbian learning, our neuromorphic robot is also equipped with computational models of place and grid cells forming a cognitive map to assist navigation in the open-field arena. In biological systems, this cognitive map is primarily attributed to specialized neurons known as grid and place cells within the medial entorhinal cortex (MEC) and hippocampus. Grid cells, located in the medial entorhinal cortex (MEC), generate a periodic hexagonal firing pattern that provides a spatial metric for navigation. These cells act as an internal coordinate system, allowing animals to track their position and direction as they move through an environment (Hafting et al., 2005, Moser et al., 2017). Place cells, found in the hippocampus, fire when an animal occupies a specific location, creating a cognitive map of the environment. Together, grid cells and place cells form a neural framework that enables rodents to navigate complex spaces, remember locations, and associate sensory cues with specific outcomes (Bush et al., 2014, Boccara et al., 2019). This biological mechanism has inspired computational models of spatial navigation, which are now being applied to neuromorphic systems to enhance robotic autonomy and adaptability in dynamic environments. Grid cells provide a multi-scale periodic representation that functions as a coordinate system. Place cells activate when rodents are in a specific location. Inspired by how rodents navigate complex environments using sensory cues, our neuromorphic robot combines visual and vibration signals to form associative learning. Unlike traditional AI, our approach requires fewer computational resources (19 neurons) while maintaining robust learning and navigation capabilities. Moreover, our associative learning model has been deployed into a neuromorphic robot and validated at both simulation and experimental scenarios. In our simulation and experimental scenarios, the visual cue is designated as a neutral stimulus (CS), while the vibration signals (US) from the road bumpers are designed to serve as aversive stimuli. This learned association enables the neuromorphic robot to proactively memorize the relationship between two concurrent events: sensing a red wall and experiencing vibration from the road bumpers simultaneously. After detecting these two stimuli several times, our neuromorphic robot memorizes the relationship between the red wall (neutral stimulus) and the vibration from the road bumper (aversive stimulus). Consequently, the neuromorphic robot evokes an avoidance movement strategy when it detects the red color with no vibration stimulus present.

The contributions of this paper are summarized as follows:

1. Replicate associative learning of rodents in an open-field arena in both simulation and experimental scenarios.
2. Integrate place and grid cell models into a neuromorphic robot and associative learning model.
3. Our associative learning model utilizes fewer neurons (19 neurons) while conducting a functional self-learning capability observed in rodents.

## 2 Development of neuromorphic robot

**FIGURE 1:**
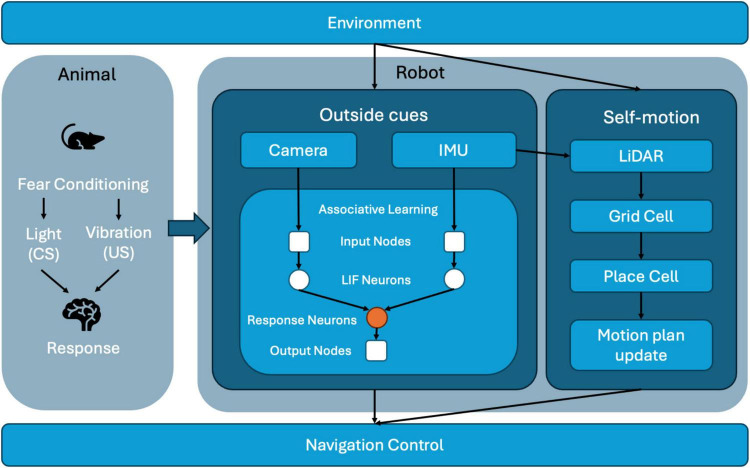
System architecture of the neuromorphic robot integrating multimodal sensory inputs, associative learning, and spatial cognition for adaptive navigation.

The proposed system architecture (Figure 1) is inspired by animal fear conditioning, a biological mechanism through which animals learn defensive responses by associating a neutral cue with an aversive stimulus. We implement a similar learning mechanism in our neuromorphic robot to dynamically adapt its navigation behavior. The robot perceives its environment through a sensor suite comprising a camera, an inertial measurement unit (IMU), and LiDAR. The camera provides visual data, analogous to animal vision, by detecting environmental cues such as colored landmarks. The IMU senses vertical acceleration, identifying vibrations that indicate unstable or hazardous terrains, analogous to proprioceptive cues in rodents. LiDAR captures spatial layout data, similar to how rodents perceive their environment through tactile feedback from their whiskers, providing real-time spatial context.

Visual and inertial signals are encoded into spiking activity by Leaky Integrate-and-Fire (LIF) neurons within an associative learning module that implements Hebbian plasticity (specifically Oja’s rule). Initially, visual cues alone have minimal influence on the robot’s behavior. However, the simultaneous detection of visual and vibration signals strengthens synaptic weights between visual input neurons and a dedicated response neuron, establishing associative memory akin to classical fear conditioning. Consequently, previously neutral visual cues trigger avoidance behaviors even in the absence of vibration. Concurrently, LiDAR-based odometry data feed a spatial cognition module, where grid and place cells create and maintain an internal cognitive map of the environment. Elevated firing rates of place cells near hazardous locations reinforce the spatial memory, further influencing navigation strategies. Ultimately, the navigation control module integrates learned associative responses with spatial mapping data, continuously adjusting the robot’s trajectory and ensuring efficient and adaptive behavior in real-time.

### 2.1 Computational model of spatial cells

In this work, we construct grid cell and place cell models that simulate spatial navigation and learning. We define grid cells with spatial and angular parameters using vector notation, transforming positions from the physical environment into a cognitive map. The simulated grid-cell models are based on interference patterns of three two-dimensional sinusoidal gratings oriented 60° apart, consistent with previous theoretical and computational studies (Boccara et al., 2019).

Our model accurately simulates neural activity in a virtual environment. Place cells are influenced by the spatial metric provided by grid cells and exhibit firing patterns associated with specific physical locations. The activity of place cells is modeled as a threshold sum of outputs from multiple grid cells. This interaction between grid and place cells ensures accurate spatial representation and navigation.

#### 2.1.1 Grid cell model

In our computational framework, grid cells are defined using vector notation:


(1) $$G_{j}=\left[s_{j},\theta_{j},\phi_{j}^{1},\phi_{j}^{2}\right],j\in Z^{+},$$

where *s_j_* is the spacing of the grid cell *G*_*j*_, θ _*j*_ ∈ [0,π/3] is the orientation of the grid cell *G_j_*. Each grid cell *j* has unique spatial and angular parameters (Zhang et al., 2024a; Zhang et al., 2024b). The phases $\phi_{j}=\left[\phi_{j}^{1},\phi_{j}^{2}\right]$ are set within the interval [0, 2π] (Bush et al., 2015, Ding et al., 2021), ensuring robust spatial representation. To make this clearer, *s_j_* represents the distance between grid firing fields, while θ_*j*_ determines the orientation of each grid field in space. The phases ϕ_*j*_ introduce unique positional shifts for each grid cell, contributing to the diversity of the spatial representation.

The transformation from the place-cell frame to the grid-cell frame is modeled by:


(2) $$\left[\begin{array}{c} x_{i}^{g}\\ y_{i}^{g} \end{array}\right]={\left(\left[\begin{array}{cc} s_{j}\,\cos\,\left(\theta_{j}\right)\,s_{j}\,\cos\,\left(\theta_{j}+\frac{\pi }{3}\right) & \\ s_{j}\,\sin\,\left(\theta_{j}\right)\,s_{j}\,\sin\,\left(\theta_{j}+\frac{\pi }{3}\right) & \end{array}\right]\right)}^{T}\,\left[\begin{array}{c} x_{i}^{p}\\ y_{i}^{p} \end{array}\right]-\left[\begin{array}{c} \vartheta_{j}^{1}\\ \vartheta_{j}^{2} \end{array}\right],$$

where the position $\left[\begin{array}{c} x_{i}^{p}\\ y_{i}^{p} \end{array}\right]$ in the place-cell frame is mapped to the grid-cell frame $\left[\begin{array}{c} x_{i}^{g}\\ y_{i}^{g} \end{array}\right],$ adjusting for preferred orientations and phase shifts. This transformation can be thought of as similar to how an animal reorients itself based on new landmarks after entering a new area. The neural activity for each position, indicated by the firing rate, is calculated as follows (Hafting et al., 2005):


(3) $$\sigma =\tan^{-1}\,\left(\kappa \,\left(\frac{d_{i}}{s_{j}}-\zeta \right)\right),$$

where *d_i_* represents the distance from the subject’s position to the grid cell’s preferred location, κ is an intensity control factor, and ζ adjusts the baseline firing rate. This equation calculates how the distance from a specific location influences the firing rate, with κ adjusting the sensitivity of the firing to distance.

When the animal moves to a new environment, external cues stimulate new place cells, forming a new local place-cell frame, denoted as *C*_2_. The grid-cell frame adjusts accordingly, adapting the firing field of the grid cell based on *C*_2_. The initial place-cell frame *C*_1_ is considered the global frame. The position transformation is modeled by:


(4) $$P_{i}^{P_{1}}=R_{P_{1}\,P_{2}}\cdot P_{i}^{P_{2}}+\pi ,$$

where $P_{i}^{P_{1}}$ represents the position in the initial place-cell frame, $P_{i}^{P_{2}}$ is the corresponding position in the current place-cell frame, and ϖ is the translation vector between these two frames. The rotation matrix *R*_*P*_1_*P*_2__ facilitates the transformation, adjusting the orientation between the two frames based on the rotation angle φ.


(5) $$R_{P_{1}\,P_{2}}={\left[\begin{array}{cc} \cos\,\left(\varphi \right)-\sin\,\left(\varphi \right) & \\ \sin\,\left(\varphi \right)\,\cos\,\left(\varphi \right) & \end{array}\right]}^{T}.$$

This rotation matrix aligns the coordinate system from the current place-cell frame to the initial frame, allowing accurate spatial analysis. This process draws directly from biological processes observed in mammals, where the hippocampus reorients itself based on changes in external cues, demonstrating the flexibility of cognitive maps.

To examine the grid cell’s firing activity, we simulated a virtual animal path by randomly walking in different virtual environments, including a circular environment with a radius of 1.3 m. The origin points of the world frame and the place-cell frame were assumed to be identical at the center of the round arena. This setup enabled us to observe and measure the grid cell’s response under controlled yet dynamic conditions, mimicking natural movement within a confined space. The grid cell used to generate the firing field is represented as:


(6) G=[1.0,π/4,0.5,0],

with hyperparameters ζ = 0 and κ = 0.5, modulating the firing activity. Higher values of κ intensify the activity around firing centers, while higher ζ values expand the firing fields, adapting the model to different environmental scales.

**FIGURE 2:**
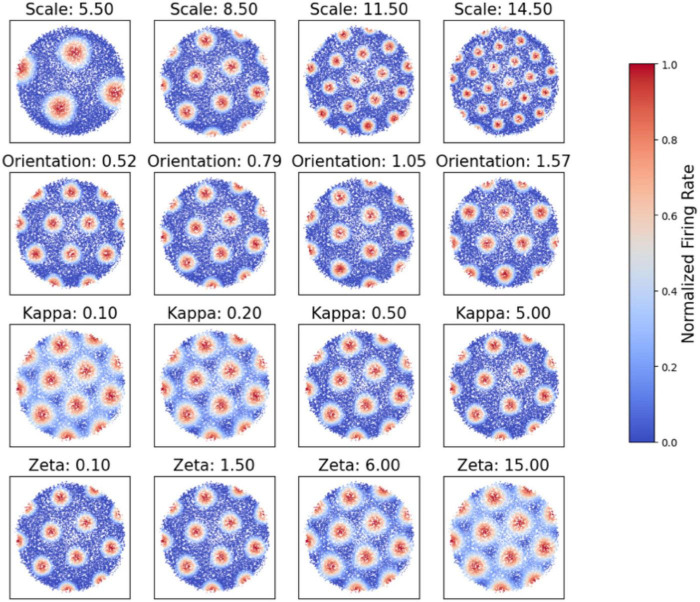
Simulation of grid cell firing patterns under varying parameters. Each subplot represents the grid cell activity under different scale (*S*), orientation (θ), kappa, and zeta values, illustrating the range of firing patterns that our model can generate.

Our simulation explores the influence of four primary parameters—scale (*s_j_*), orientation (θ_*j*_), κ, and ζ —on the emergence and structure of grid cell firing fields. Adjustments in these parameters result in more pronounced hexagonal patterns characteristic of grid cells. Figure 2 demonstrates the flexibility of our model by showing how varying κ and ζ affect firing activities. Each subplot represents grid cell activity under different parameter values, illustrating the range of firing patterns our model can generate.

#### 2.1.2 Place cell model

Place cells in the hippocampus are critical for spatial navigation and memory formation. These neurons exhibit firing patterns distinctly associated with specific physical locations within an environment. A place cell fires most strongly when the subject is at a particular area, known as the cell’s “place field.” The firing intensity of these cells decreases as the subject moves away from this central location. This unique firing characteristic ensures that each place cell responds optimally at different locations, creating a detailed spatial map within the brain.

The activity of place cells is influenced by inputs from grid cells, which provide a regularized spatial metric. The interaction between place cells and grid cells can be modeled as follows:


(7) $$P_{c\,\left(t\right)}=\Theta \,\left(\sum_{i=1}^{N}G_{C_{i}\,\left(t\right)}\right),$$

where *P*_*c*(*t*)_ represents the activity function of place cells at time *t*, Θ is a step function, and *G*_*C*_*i*_(*t*)_ denotes the activity of the *i^th^* grid cell. This equation implies that the place cell activity is a thresholded sum of the outputs from multiple grid cells, each contributing to the overall spatial representation in the hippocampus.

#### 2.1.3 Interaction between grid and place cells

This interaction is crucial for supporting associative learning in neuromorphic systems. By associating specific sensory cues (e.g., visual or tactile signals) with spatial representations, the system can learn to predict outcomes based on past experiences. This capability mirrors biological associative learning processes and enhances the neuromorphic system’s adaptability.

To navigate and map its environment effectively, the hippocampal system utilizes visual landmarks as positional references, which are integrated into the neural representation of space through the following response function:


(8) $$L\,P_{i}=\sum e\,x\,p\,\left[-\frac{{\left(d_{i}\,\left(t\right)-d_{i}^{k}\,\left(t\right)\right)}^{2}}{\partial_{d}^{2}}-\frac{{\left(\theta_{i}\,\left(t\right)-\theta_{i}^{k}\,\left(t\right)\right)}^{2}}{\partial_{\theta }^{2}}\right].$$

The *LP*_*i*_ represents the activity of a place cell *i*, which encodes the position of an individual within the environment. It quantifies how strongly the cell responds to the current location based on its preferred spatial properties. Higher values of *LP*_*i*_ indicate a closer match to the cell’s preferred location, making it a key measure of the cell’s contribution to spatial representation. In this model, *d*_*i*_(*t*) and θ_*i*_(*t*) represent the distance and angle of the *i^th^* landmark relative to the subject, respectively. At the same time, $\partial_{d}^{2}$ and $\partial_{\theta }^{2}$ are variance terms that adjust the sensitivity of the response to positional discrepancies. This function ensures that the spatial memory is updated accurately by adjusting for perceptual errors and discrepancies between remembered and observed landmark positions.

The integration of vibrational cues into the firing mechanisms of place cells, supported by the structured input from grid cells, offers a robust framework for understanding spatial cognition. By adapting the neural responses based on environmental stimuli and correcting for navigational errors using landmark recognition, this model underscores the dynamic nature of spatial memory and its critical role in adaptive behavior.

### 2.2 Perception of neuromorphic robot

We utilized the Gazebo simulation platform to simulate grid and place cell behaviors in the neuromorphic robot. Gazebo is a widely used open-source robotics simulator that provides high-fidelity physics simulation, sensor modeling, and dynamic environment interaction, making it ideal for testing and validating robotic systems in realistic scenarios (Koenig and Howard, 2004). Its integration with the Robot Operating System (ROS) enables seamless communication between simulated sensors, actuators, and control algorithms, facilitating the development of complex robotic behaviors. Its high fidelity in modeling physics and dynamic interactions provides an ideal environment for accurately simulating navigation tasks and sensory scenarios. Its integration with the ROS (Robot Operating System) framework ensures that sensor inputs and movement responses closely resemble real-world conditions.

In conjunction with Gazebo, we employed Rviz, a real-time visualization tool, to analyze and adjust the grid and place cell models immediately. Rviz (ROS Visualization) is a powerful 3D visualization tool integrated with the Robot Operating System (ROS). It allows users to visualize sensor data, robot models, and environmental maps in real time, making it an essential tool for debugging and developing robotic systems (Quigley et al., 2009). In our work, Rviz was used to monitor the robot’s LiDAR data, odometry, and navigation paths, providing immediate feedback for model validation and adjustment. The red points in Rviz indicate the Light Detection and Ranging (LiDAR) boundary detection, which is limited to 270 degrees, while the green line traces the robot’s path using odometry data.

**FIGURE 3:**
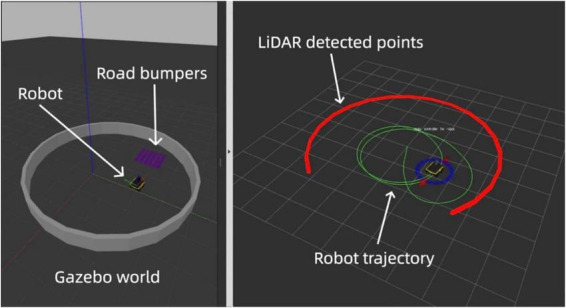
Simulated and real-time visuals of the neuromorphic robot’s path in Gazebo (left) and Rviz (right). The red line in Rviz shows LiDAR-detected boundaries with gaps indicating its 270-degree range. The green line traces the robot’s path via odometry.

Figure 3 displays our comprehensive simulation setup, showcasing the integration of Gazebo for environment modeling and Rviz for dynamic data visualization, which together form a robust platform for developing and testing advanced navigational strategies based on neuromorphic grid and place cell models.

The cell-based navigation system aims to enhance the interaction between the robot and its environment through an associative learning model. We integrated visual and vibration signals, enabling the robot to connect known and unknown cues, such as conditional and unconditional stimuli, in associative learning. Inspired by biological learning mechanisms, this model strengthens relationships between multiple sensory inputs, allowing the robot to adapt autonomously to dynamic environments.

Visual and vibration stimuli are often vital cues in rodent experiments. In our neuromorphic robot, visual signals from a stereo camera provide critical environmental information, while vibration signals from the accelerometer reflect physical interactions such as terrain changes or collisions. The robot can learn from past experiences and develop predictive responses by associating specific visual patterns with vibration signals.

Our experiments aimed to validate the computational models by simulating the grid and place cell behaviors in a neuromorphic robot navigating a controlled circular arena. This setup allowed us to emulate the free movement of a rat and observe the robot’s behavior in an environment where biological grid cells are known to generate hexagonal firing fields. By drawing parallels to animal behavior, we aimed to understand how well these neuromorphic models perform in real-world scenarios and how effectively they generate biologically inspired spatial representations. Using the robot’s navigation system, we set the primary grid cell model parameters to:


(9) $$G=\left[8.8,\frac{\pi }{4},0.5,1.2\right].$$

we modulated the firing rate’s range and intensity with ζ = 0.3 and κ = 5.0. Concurrently, place cell models were integrated to process vibrational data, providing additional environmental context for spatial memory and navigation. The vibration analysis, particularly when encountering road bumpers, further refined the place cell response, enhancing the robot’s obstacle detection and navigation acumen.

**FIGURE 4:**
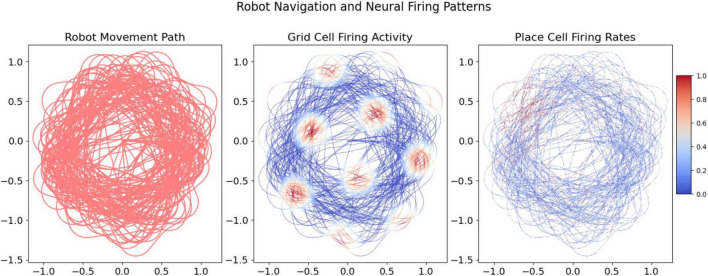
Visualization of the robot’s movement paths and the corresponding neural firing activities over increasing data points. The grid and place cell firing activities provide insight into the robot’s spatial exploration and the computational model’s response.

The navigational paths and neural activity, as depicted in Figure 4, demonstrate the robot’s ability to replicate the characteristic hexagonal pattern of biological grid cells. This also validates the integration of place cell models informed by vibrational cues. The dual modeling approach provides robust empirical support for the computational navigation system, emphasizing its accuracy and relevance to spatial navigation tasks.

#### 2.2.1 Vibration signal processing

Vibration signal is a crucial indicator for detecting vertical movements, impacts, and changes in terrain. Vibration was chosen as the unconditional stimulus (US) due to its saliency, biological relevance, and practicality. Vibration provides immediate and unambiguous feedback about physical interactions, such as collisions or uneven terrain, making it ideal for triggering avoidance behavior (Kandel et al., 2000). Additionally, vibration mimics the tactile feedback rodents rely on for navigation, such as whisker or paw interactions (Diamond et al., 2008). From a practical perspective, vibration is easily measured using accelerometers in IMUs, which are standard in robotic systems, ensuring efficient and reliable integration (Goodfellow, 2016). It acts as an automatic response mechanism akin to the innate recognition of danger in animals like rats. For this reason, vibration is treated as the unconditional stimulus in our model.

The robot’s accelerometer constantly monitors movements by capturing acceleration data along three axes (x, y, and z). This study focuses on the *z*-axis because it is most sensitive to vertical displacements, such as encountering bumps, drops, or uneven terrain. When the robot runs over road bumps or similar irregularities, the acceleration along the *z*-axis changes rapidly, resulting in detectable vibrations. To quantify these vibrations, we compute the total vibrational force using the following formula:


(10) $$a=\sqrt{x^{2}+y^{2}+{\left(z-g\right)}^{2}},$$

where *g* approximates the gravitational acceleration constant at 9.81*m*/*s*^2^. This calculation provides a scalar magnitude of the vibrational force exerted on the robot due to irregularities in the surface texture and obstacles. The IMU measures acceleration along three axes (x, y, and z), but we focus primarily on the *z*-axis for vibration detection because it is most sensitive to vertical displacements, such as bumps encountered by the robot. The x and y axes, which capture lateral and longitudinal movements, are less relevant for detecting vertical vibrations and are therefore not used in this analysis.

To process the raw IMU data, we apply a low-pass filter to reduce high-frequency noise, which is common in dynamic environments due to factors such as motor vibrations, sensor sensitivity, and external disturbances. The low-pass filter is designed to preserve meaningful vibration signals while attenuating high-frequency noise, ensuring that the robot responds only to significant tactile events. After filtering, the *z*-axis data is further smoothed using a moving average filter to reduce sharp fluctuations and produce a stable signal. This preprocessing ensures that the vibration data is clean and suitable for neural processing, enabling the robot to reliably detect and respond to significant tactile events.

Here, *g* represents the gravitational acceleration constant, approximately 9.81*m*/*s*^2^. This adjustment accounts for the deviation of the vertical acceleration from the standard gravitational acceleration, highlighting changes from the expected pull of gravity. In a perfectly steady environment, the vertical acceleration z should equal g. However, when the robot encounters vertical disturbances, such as bumps or drops, the value of z deviates from g, creating the observed vibration. This formula thus provides a scalar magnitude of the vibrational force, capturing the effects of surface irregularities on the robot’s mobility.

**FIGURE 5:**
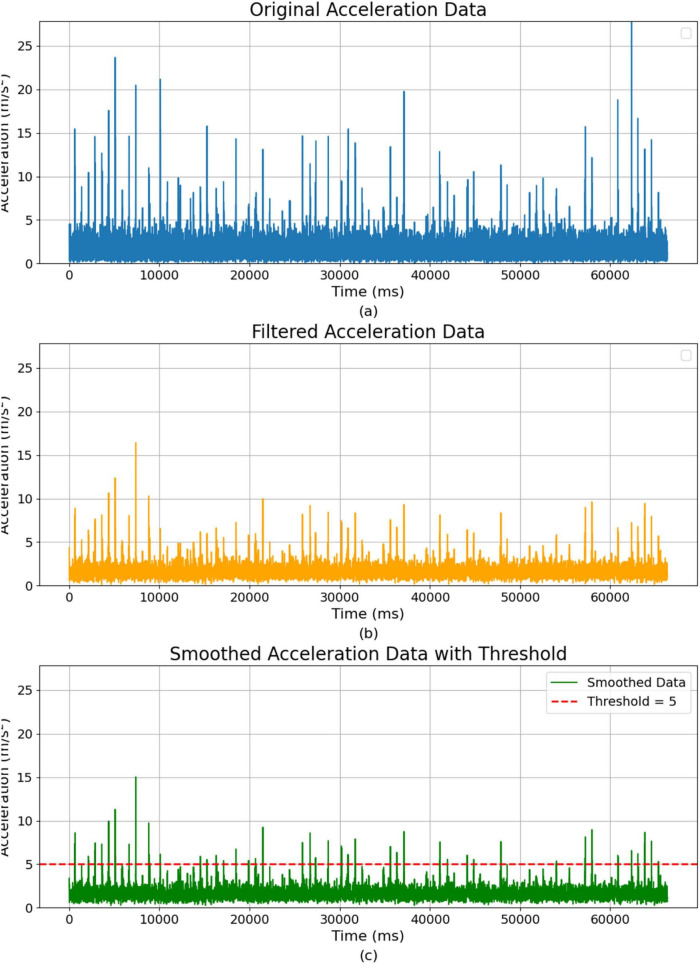
Acceleration data over time: raw, filtered, and smoothed representations. **(A)** Original acceleration data. **(B)** Filtered acceleration data after noise reduction. **(C)** Smoothed acceleration data with a threshold.

In Figure 5A, the raw *z*-axis data from the inertial measurement unit (IMU) often contains noise due to the sensor’s sensitivity and the dynamic nature of the robot’s environment. This noise arises from several factors, including external environmental influences, such as rough terrain or sudden impacts, which cause abrupt changes in acceleration. The IMU sensor is also susceptible to detecting small vibrations from sources like the robot’s motors, thermal fluctuations, or electromagnetic interference. The robot’s movement dynamics, such as rapid changes in speed or direction, can also contribute to unwanted fluctuations in the data. These various forms of noise can mask the meaningful signals related to the robot’s vertical movements, making it necessary to preprocess the data before using it in the neural network. We apply several preprocessing steps to prepare the *z*-axis vibration data for neural processing.

As shown in Figure 5B, the first step is noise reduction, the raw IMU data is often contaminated with high-frequency noise. To reduce this noise, we apply a low-pass filter, which removes frequencies above a certain threshold while preserving the lower-frequency components more indicative of significant vertical movements. In our experimental setup, vertical (*z*-axis) acceleration dominated the vibration signal, justifying the focus of the analysis on this axis. Bumps and uneven ground produced significantly larger acceleration spikes in the vertical direction compared to the x or y directions. This finding aligns with prior studies indicating vertical accelerations as the most significant indicators of rough terrain. We also analyzed horizontal (*x* and *y*-axis) accelerations to check for cross-axis correlations, and finding them to be minimal and inconsistent during the experiment. As a result, lateral motions provided little additional information and did not enhance the detection of hazardous vibrations. Thus, sensor fusion of *x*- and *y*-axis data with *z*-axis data was unnecessary, allowing our analysis to focus exclusively on vertical acceleration signals. This filtering process can be represented as:


(11) $$a_{z,f\,i\,l\,t\,e\,r\,e\,d}\,\left(t\right)=\int_{-\infty }^{t}e^{-\frac{\left(t-\tau \right)}{\tau_{l\,p}}}\,a_{z}\,\left(\tau \right)\,d\tau ,$$

where *a*_*z*,*filtered*_(*t*) is the filtered *z*-axis acceleration, and τ_*lp*_ is the time constant of the low-pass filter, which controls the cutoff frequency.

All IMU acceleration signals were processed through a low-pass filter to remove high-frequency noise while preserving critical low-frequency vibration signatures related to terrain irregularities. We selected a cutoff frequency of approximately 5 Hz, which corresponds to a filter time constant of roughly 0.2 s. This specific frequency was chosen because typical significant vertical acceleration events caused by the robot traversing bumps or obstacles occur primarily at lower frequencies (around 1–4 Hz). Thus, the 5 Hz cutoff frequency ensures that relevant motion signals are clearly captured, while higher-frequency noise and minor mechanical vibrations are effectively suppressed. The chosen filter parameters balance signal integrity with noise reduction, aligning precisely with the robot’s motion characteristics and the environmental conditions encountered during experiments.

The second step is smoothing, Figure 5C shows the vibration data can exhibit sharp fluctuations that may not be relevant to the system’s overall behavior. We apply a moving average filter to smooth these fluctuations, which averages the acceleration values over a specified time window, producing a more stable signal. The smoothed signal is given by:


(12) $$a_{z,s\,m\,o\,o\,t\,h\,e\,d}\,\left(t\right)=\frac{1}{N}\,\sum_{i=0}^{N-1}a_{z,f\,i\,l\,t\,e\,r\,e\,d}\,\left(t-i\right),$$

where *N* is the size of the moving window. The resulting smoothed and filtered *z*-axis data is then normalized to ensure that it fits within a suitable range for neural processing. This normalization process scales the data to a fixed range, typically between 0 and 1, ensuring consistent input to the neural network regardless of the magnitude of the raw data.

After preprocessing, the vibration data is normalized and integrated into the neural network for further processing. To handle this data, we utilize a Leaky Integrate-and-Fire (LIF) neuron model (Abbott, 1999), which is well-suited for simulating the spiking behavior of neurons in response to time-varying input signals such as vibration data. The LIF model is particularly suitable for this task because it integrates time-varying inputs over time and generates discrete spikes when the membrane potential exceeds a defined threshold.

These spikes provide an event-driven representation of the vibration signal, mimicking the way biological neurons process and transmit information. The behavior of the LIF neuron is governed by the differential equation:


(13) $$\tau_{m}\,\frac{d\,V\,\left(t\right)}{d\,t}=-\left[V\,\left(t\right)-V_{r\,e\,s\,e\,t}\right]+R\,I\,\left(t\right),$$

where *V*(*t*) represents the membrane potential at time *t*, *V*_*reset*_ is the resting membrane potential, τ_*m*_ is the membrane time constant, *R* is the membrane resistance, *C* is the membrane capacitance, and *I*(*t*) is the input current to the neuron. The resting potential *V*_*reset*_ is set to 0 mV to ensure that the neuron starts from a baseline state after each spike. The membrane time constant, defined as τ_*m*_ = *RC*, which determines the rate at which the membrane potential decays without input. For this model, the time constant τ_*m*_ is set to 0.02 s, allowing the neuron to respond to transient input signals rapidly. This value ensures that the neuron is sensitive to abrupt changes in the vibration data, which indicate significant tactile events. The membrane resistance is calibrated to balance the neuron’s sensitivity and stability, ensuring that the neuron reacts appropriately to meaningful inputs without spiking excessively due to noise. The input current represents the processed vibration data after normalization, scaling the input to a consistent range suitable for neural processing.

A key parameter for the vibration LIF neuron is the firing threshold, which determines the membrane potential required to generate a spike. For this model, the threshold corresponds to a vibration magnitude of 5 *m*/*s*^2^ or greater. This value reflects the observation that a magnitude of 5 *m*/*s*^2^ signifies significant vertical movements or surface irregularities, such as bumps or drops encountered by the robot. By setting the firing threshold to align with this magnitude, the neuron spikes only in response to meaningful tactile events, filtering out minor fluctuations and noise in the data. The refractory period is set to 0.002 s, allowing the neuron to quickly reset after a spike and remain responsive to subsequent input signals.

After being filtered and smoothed, the normalized vibration data serves as the input to the vibration LIF neuron. This preprocessing ensures that the vibration LIF neuron reacts more accurately to meaningful changes in the vibration intensity rather than to high-frequency noise. When the vibration magnitude exceeds the threshold, the neuron’s membrane potential crosses the firing threshold, resulting in a spike. The spiking activity encodes significant tactile events as discrete spikes, with higher spike frequencies corresponding to more intense or abrupt changes in the vibration signal.

The single LIF neuron provides a biologically plausible mechanism for representing tactile information by encoding the vibration input as a time-varying spike train. This spike-based representation captures significant changes in the vibration intensity while ignoring irrelevant noise, ensuring that only meaningful variations in the input signal are processed. In the next stage, the spikes generated by the vibration-sensitive neuron are combined with visual spikes in the associative learning model. During training, the simultaneous spiking of vibration and visual neurons strengthens the synaptic weights associated with the visual input. This process enables the robot to respond to visual input alone after training, even in the absence of vibration, simulating the acquisition of a conditioned response.

**FIGURE 6:**
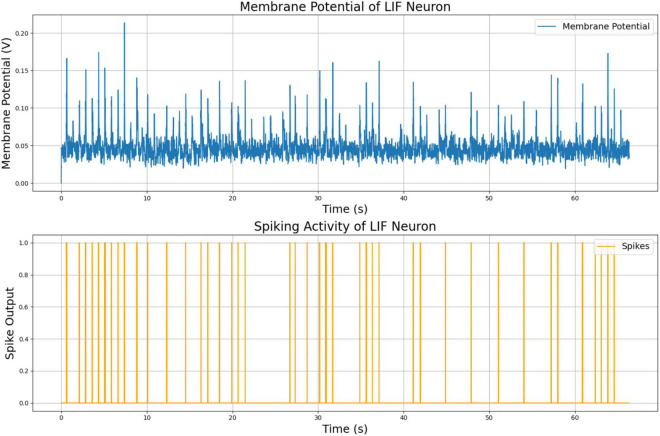
Membrane potential and spiking activity of vibration LIF neuron model.

The membrane potential and spiking activity of the vibration-sensitive LIF neuron are shown in Figure 6. These plots illustrate the neuron’s ability to respond to variations in the vibration magnitude, producing spikes in response to abrupt or significant changes in the input signal. This functionality demonstrates the model’s capacity to capture and encode tactile information discretely, event-driven, providing a reliable mechanism for robust sensory integration and learning.

#### 2.2.2 Visual signal processing

Color signals play a crucial role in our model, particularly in identifying and reacting to specific visual cues, such as the presence of red objects in the environment. While particular colors may not inherently hold significance for rats as unconditional stimuli, associative learning enables the model to give meaning to colors like red when combined with vibration data as the degree of association increases.

**FIGURE 7:**
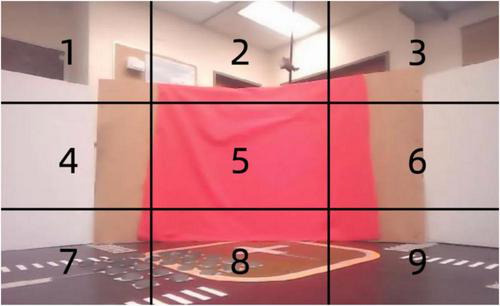
Spatially segmented visual input for associative learning: red pixel detection across nine regions.

A stereo camera mounted on the robot captures visual signals and divides each image into nine distinct regions as shown in Figure 7. Each of these regions is then processed independently to extract relevant color information, specifically focusing on the detection of red. Red may indicate critical environmental features, such as obstacles or navigation markers. These nine regions are fed into different neurons, each responsible for processing color data from its designated area.

Each neuron receives visual input from its assigned region and integrates the color intensity over time. The neurons are implemented as LIF neurons, with parameters tuned to match the temporal dynamics of the visual input. The membrane time constant is set to τ_*m*_ = 0.05, allowing the neurons to integrate color signals over a relatively long period. This ensures that transient red color detections do not result in spurious spikes. The refractory period τ_*ref*_ = 0.002, providing that the neurons recover quickly enough to respond to sustained visual stimuli while avoiding excessive spiking.

When a neuron’s membrane potential exceeds a predefined threshold, it fires a spike, indicating that significant red color detection has occurred in that particular region. This spike suggests the presence of red color in one of the nine regions monitored by the camera. The system can spatially localize the red color within the robot’s environment by having each of the nine neurons handle a specific region.

The spikes generated by the neurons from the nine regions serve as input to the associative learning model, as shown in Figure 7. These visual inputs are represented as a vector, with each element corresponding to the spiking activity of one of the color LIF neurons responsible for its region. This high-dimensional vector captures the spatial distribution of color in the environment, enabling the neural network to associate visual patterns with corresponding vibration signals for tasks such as navigation or object recognition.

The red color input to the model is derived by dividing the visual field captured by the robot’s camera into nine equal regions, as illustrated in the Figure 7. For each region, the proportion of red pixels to the total number of pixels is calculated, providing a measure of the intensity of red in that area. This measure, referred to as the red pixel rate, is used as the input signal to the color neurons. Specifically, the red pixel rate for a given region is computed as the ratio of the number of red pixels detected to the total number of pixels within that region.

Each red pixel rate is then normalized to ensure consistent scaling across all regions and is provided as input to a corresponding color LIF neuron. The color LIF neurons process these normalized red pixel rates, converting the continuous input into spiking activity based on the neuron’s firing threshold and other parameters. This spiking activity captures the presence and intensity of red color in the respective regions over time, providing a biologically inspired representation of the visual input. By dividing the visual field and processing each region independently, the model achieves spatial localization of visual features, allowing the neural network to learn associations between specific areas of the visual field and corresponding tactile stimuli.

This structured input processing ensures that the model can effectively detect and react to red color patterns, with the spatially distributed spiking activity serving as a robust input representation for the associative learning model. This design enables the robot to leverage spatially localized visual information to enhance its ability to navigate and recognize objects in dynamic environments.

**FIGURE 8:**
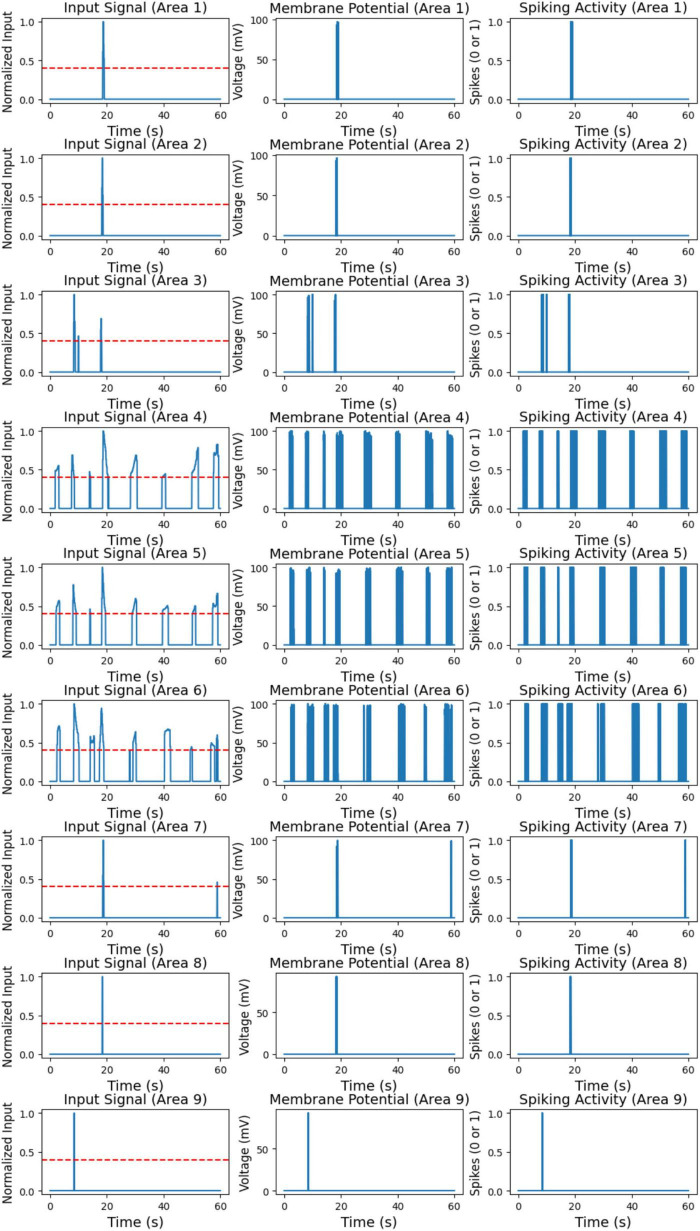
Processed input signal for nine areas (left). Membrane potential of color LIF neurons for nine areas (middle). Spiking activity of color LIF neurons for nine areas (right).

In Figure 8 (left), the processed input signal for each of the nine areas over the 60-s simulation is shown. This signal represents the red pixel rates detected in each area, normalized to capture only significant red pixel presence periods. A threshold of 0.4 was applied to the normalized data, meaning that only red pixel rates above this threshold were retained; values below the threshold were set to zero. This processing step was essential to prevent neuron activation due to noise or low red pixel rates, ensuring that only meaningful red pixel detections were passed to the neuron model.

The input signals in Figure 8 (left) vary across different areas, highlighting the spatial distribution of red pixel detections. Certain areas, such as Area 1, Area 7, and Area 8, exhibit isolated peaks in the input signal, indicating sporadic but significant red pixel detections. In contrast, areas like Area 4, Area 5, and Area 6 display more frequent high input signals, suggesting consistent red pixel detections at regular intervals throughout the simulation. Some areas, such as Areas 2 and 3, show minimal or no high input signals, indicating little to no red pixel detection in these regions. Overall, the processed input signals effectively isolate significant red pixel detection periods for each area, providing a clear and noise-free input to the neuron model.

Figure 8 (middle and right) shows the membrane potential and spiking activity of the color LIF neurons corresponding to each of the nine areas. The membrane potential plots on the left depict the voltage response of each neuron to the input signals. In contrast, the spiking activity plots on the right indicate moments when each neuron fires, represented as a binary output (0 for no spike, 1 for spike). These plots illustrate the neuron model’s response to high red pixel rates, highlighting the relationship between input intensity and neuron activation.

The neurons exhibit distinct responses depending on the frequency and intensity of the input signals. In areas with intermittent high input signals, such as Area 1, Area 7, and Area 8, the neurons show occasional spikes corresponding to the brief input peaks observed in the processed signal. Outside these peaks, the neurons remain at resting potential, demonstrating that they respond selectively to high red pixel rates while remaining inactive without significant input. In areas with frequent and sustained high input, such as Area 4, Area 5, and Area 6, the neurons display prolonged periods of elevated membrane potential and consistent spiking. This continuous activation aligns with the consistent red pixel detections in these areas, as shown in the input signal. For areas with minimal input signal, such as Area 2 and Area 3, the neurons remain at resting potential with little to no spiking activity. This indicates that the neuron model effectively ignores low or insignificant input values, as intended.

These results demonstrate that the color LIF neuron model responds selectively to high red pixel rates, spiking only when the input exceeds the predefined threshold. The processed input signal successfully filters out low red pixel rates, ensuring neurons activate only in response to significant stimuli. This selective response reduces noise and simulates biologically realistic behavior in neuromorphic models, validating the model’s effectiveness in detecting and responding to meaningful visual stimuli in dynamic environments.

#### 2.2.3 Integration of sensory inputs

The integration of sensory modalities in our system is achieved through a biologically inspired neural framework, where visual and tactile signals are encoded as spiking activity and combined using Hebbian learning to form associative representations. This section details the technical mechanisms by which the neuromorphic robot processes and merges camera and IMU signals to generate adaptive behavior.

Visual signals are captured by the stereo camera and divided into nine predefined spatial regions. For each region, the red pixel intensity is calculated and normalized. These values are then fed into nine separate Leaky Integrate-and-Fire (LIF) neurons, each responsible for one region. The membrane potential of each LIF neuron integrates the incoming red intensity over time, producing spikes when the accumulated potential exceeds a defined threshold. This design ensures spatial and temporal encoding of red cues, simulating the processing of salient visual stimuli. Meanwhile, vibration signals from the IMU are focused on the *z*-axis, capturing vertical acceleration deviations indicative of surface irregularities. After filtering and smoothing the raw data (as described in Section 2.2.1), the signal is normalized and provided as input to a dedicated vibration-sensitive LIF neuron. This neuron also encodes the magnitude of tactile events as spike trains, firing only when the vibration intensity surpasses a threshold (set at 5 m/s^2^ in our implementation).

The associative learning module receives the spiking output from both the color LIF neurons (Conditioned Stimuli, CS) and the vibration LIF neuron (Unconditioned Stimulus, US). These inputs converge on a shared response neuron, which integrates the co-occurrence of spikes. Initially, the synaptic weights from the vibration neuron to the response neuron are set high, ensuring immediate reaction to tactile danger. In contrast, the visual pathway has low initial weights, preventing the robot from reacting to color alone. Over time, when both visual and vibration spikes are detected simultaneously, the synaptic weight from the visual input is increased using Oja’s rule, a stabilized version of Hebbian learning. This weight update mechanism enables the robot to gradually learn to associate visual cues with potential tactile danger.

To ensure real-time operation, all inputs are processed frame by frame in synchrony. The system utilizes ROS to synchronize IMU and camera data streams, aligning their timestamps to guarantee accurate correlation of multimodal inputs. The spiking outputs of the neurons are evaluated during the experiment which enabling the robot to react promptly to learned associations. Once the learned synaptic weight for a visual input exceeds a threshold, red alone is enough to activate the response and trigger the output neurons. This output is interpreted as an avoidance command and is relayed to the navigation control system. The robot then performs an avoidance maneuver (e.g., backward movement or rotation) until the red cue is no longer in its field of view, while the spatial memory system, constructed from grid and place cells, contributes to environmental context. Place cells that represent locations near high-vibration zones become highly active, reinforcing the robot’s decision to avoid those areas even if the visual cue is ambiguous. This integrated sensory and spatial processing pipeline allows the robot to make informed, adaptive decisions in real-time.

**FIGURE 9:**
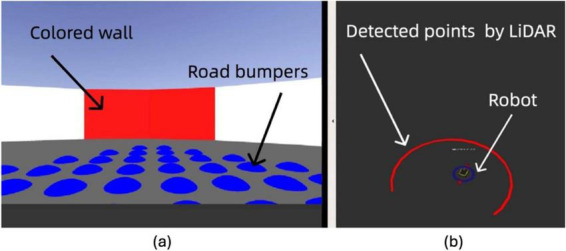
Simulation of robot perception: **(A)** camera view. **(B)** Overhead view with LiDAR detection.

In Figure 9, the robot encounters a scenario with a red wall as a visual cue and simulated ground vibrations. This test environment assesses the robot’s capability to prioritize visual information in decision-making processes, remarkably when vibrations suggest an uneven area. The associative learning model adjusts the weight of visual cues in the robot’s navigational method, reflecting an increased reliance on visual information when vibrations are detected.

**FIGURE 10:**
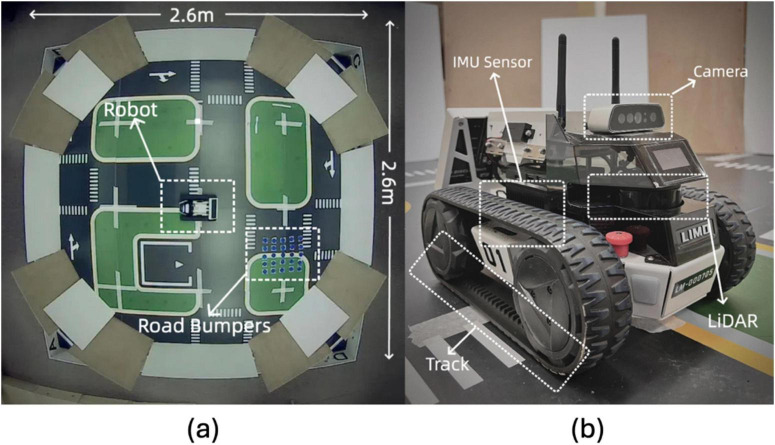
Experimental setup and robot hardware configuration: **(A)** Overhead view of the 2.6 m × 2.6 m testing environment with road bumpers and the robot. **(B)** Close-up of the LIMO robot with labeled sensors, including IMU, camera, LiDAR, and track system.

The real-world experiments were conducted in a controlled arena to simulate features and obstacles. As depicted in Figure 10, the arena spans 2.6 m in diameter, with the neuromorphic robot starting at the center each time. Road bumpers are placed to test the robot’s navigation and sensory processing capabilities in a complex setting. The layout includes various navigational challenges and is annotated with dimensions and critical elements, such as the neuromorphic robot and road bumpers, to provide a scale and context.

Our real-world experimentation involved evaluating the neuromorphic robot’s navigational method within an open field arena with high walls detectable by the robot’s LiDAR system. This setup provided a continuous boundary simulating the operational environment that grid and place cells theorize to navigate. The testing emphasized the robot’s ability to utilize its onboard sensors for orientation and navigation in environments that mimic real-world scenarios. We optimized the robot’s power management systems and adapted operational strategies to address challenges such as energy constraints and data limitations encountered during these tests.

### 2.3 Motion plan of neuromorphic robot

The neuromorphic robot combines data from LiDAR and IMU to achieve precise path planning and obstacle avoidance. LiDAR offers detailed environmental mapping, while IMU supplies movement dynamics and orientation data. These sensory inputs enhance the robot’s real-time trajectory planning, ensuring safe navigation through complex environments.

The neuromorphic robot integrates LiDAR and IMU data to achieve precise path planning and obstacle avoidance, relying on a structured movement logic that continuously adapts based on sensory feedback. The process begins with the robot entering an initial “Move” state, from which it continues looping as long as the ROS is active. This design enables the robot to dynamically respond to changing environmental inputs without requiring manual intervention. The robot movement logic is shown in Algorithm 1.

**ALGORITHM 1:** Movement Logic of the Robot’s Self-Navigation

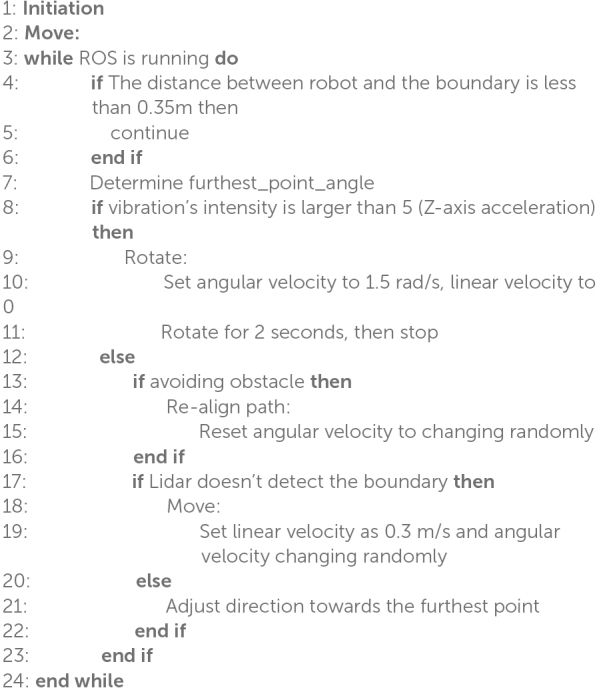

Within this loop, the LiDAR is used to detect boundaries. When no boundary is identified, the robot proceeds without altering its path. However, if a boundary is detected, the robot determines the direction of the furthest point and plans an optimal escape route around obstacles. Additionally, the IMU continuously monitors vibrations to identify irregular surfaces or obstacles. Upon detecting vibration, the robot initiates a rotation, setting its angular velocity to 1.5 rad/s while reducing its linear velocity to zero. This rotation continues for 2 s, allowing the robot to evaluate its surroundings and adjust its orientation as necessary.

After rotating, the robot checks whether it has successfully avoided the obstacle. If so, it re-aligns its path and resets the angular velocity to its original state, ensuring that it can regain its intended direction. The robot then uses the LiDAR to re-check for boundaries; if no boundary is detected, it moves forward, introducing random variations in angular velocity to prevent it from becoming stuck in repetitive patterns. When a boundary is detected, the robot adjusts its direction toward the furthest detected point, ensuring it moves toward open space.

**FIGURE 11:**
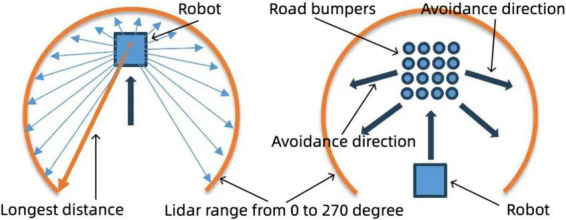
Robot navigation strategy: boundary and road bumper avoidance. Left: Boundary avoidance using LiDAR data, selecting the longest distance for navigation within a 0–270° range. Right: Road bumper avoidance strategy, adjusting movement direction based on detected obstacles.

The robot continuously adjusts its linear velocity and direction until a clear path is found, repeating this process to maintain efficient navigation while avoiding obstacles. This movement logic centers around the seamless integration of LiDAR and IMU data, allowing for robust obstacle detection and avoidance. The LiDAR maps the environment and identifies boundaries like Figure 11 (left), while the IMU detects surface irregularities through vibrations like Figure 11 (right). The combination of these inputs enables the robot to make informed decisions on when to rotate, adjust velocity, or proceed along a clear path. By alternating between these states, the robot effectively plans its trajectory, navigates obstacles, and maintains a safe and efficient movement pattern through complex environments. The inclusion of angular velocity adjustments and random movement variations helps ensure that the robot does not become stuck, allowing it to continuously explore even challenging terrains.

## 3 Associative learning using the neuromorphic robot

### 3.1 Integration of visual and vibration inputs

During our experiments, the robot was programmed to alter its path whenever the detected vibration exceeded a threshold value of 5 *m*/*s*^2^. This threshold was determined based on preliminary tests identifying vibration intensities characteristic of risky areas, such as near road bumpers or uneven terrain.

**FIGURE 12:**
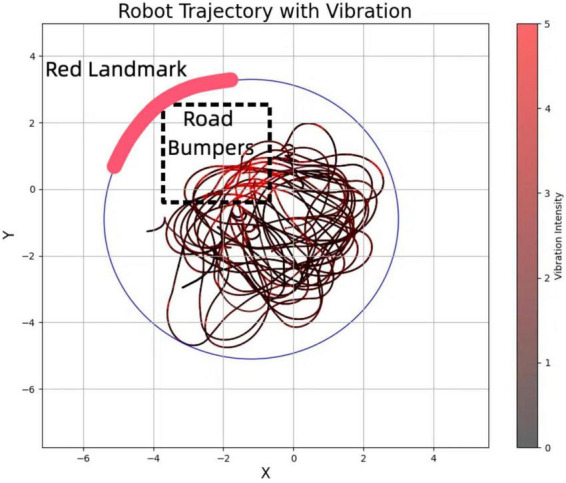
The trajectory shows the robot avoiding areas with vibration, marked as “Road Bumpers.” The avoidance behavior is triggered when vibration is detected, prompting the robot to reroute.

As depicted in Figure 12, the robot effectively avoided entering high-vibration zones. The plotted trajectory illustrates how the robot approaches these zones but re-routes upon detecting high vibration, thereby avoiding the “Road Bumpers” area. This behavior highlights the robot’s dynamic response to sensory inputs and its potential to enhance safety and operational efficiency in autonomous navigation.

Our approach to enhancing the robot’s navigational capabilities involved developing an associative learning algorithm that integrates visual and vibration sensory inputs. This algorithm enables the robot to learn from environmental interactions by associating specific colors with vibration levels. As the robot explores, it learns to recognize and react to environmental cues that indicate potential hazards or areas of interest.

During the initial phases of exploration, the robot uses its camera to detect specific colors associated with different terrain textures or obstacles. Simultaneously, the vibration sensors measure the intensity of ground vibrations, which correlate with different types of surfaces (e.g., road bumpers).

When the robot encounters high vibration intensities, it associates these vibrations with visual cues at those locations. Over time, through repeated exposure and feedback, the robot learns to predict potential obstacles or uneven terrain based solely on visual information, even when vibrations are not present.

### 3.2 Hebbian learning and Oja’s rule

**FIGURE 13:**
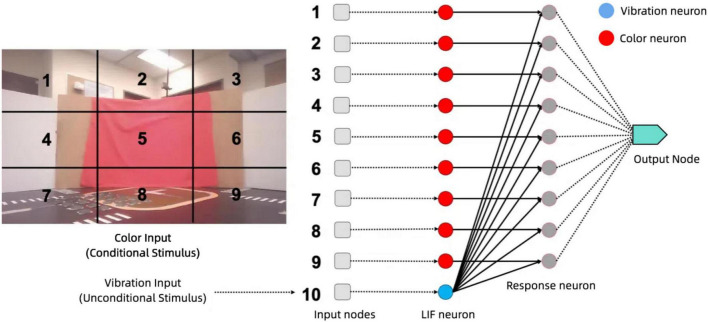
Associative learning model architecture: processing visual and vibration inputs through LIF neurons, Hebbian Learning, and response generation.

The core of our approach lies in the associative learning model in Figure 13, which integrates visual and vibration signals to enable the robotic system to learn and respond to complex environmental stimuli. By employing Hebbian learning principles through Oja’s rule, the model strengthens connections between neurons co-activated by these sensory inputs, allowing the system to anticipate and adapt to situations based on past experiences and interactions with the environment.

This associative learning model is implemented in Nengo as a network of LIF neurons. Nengo is a neural simulator and development framework that enables the design, simulation, and deployment of large-scale neural models. It supports a wide range of neuron models, learning rules, and hardware backends, making it a versatile tool for neuromorphic computing research (Bekolay et al., 2014). In our work, Nengo was used to implement the associative learning model, leveraging its support for Leaky Integrate-and-Fire (LIF) neurons and Hebbian learning mechanisms. It is structured to process inputs from visual and vibration sources and integrate them for a cohesive understanding of the environment. The visual processing stage consists of nine input nodes, each corresponding to a distinct region of the visual field captured by the robot’s camera. Each node feeds into a dedicated LIF neuron that processes the color information within its assigned region. These neurons are tuned to detect the presence of red, a significant environmental feature, and output spiking activity that reflects the presence or absence of red in their region. The vibration processing stage consists of a single vibration LIF neuron that receives input from the *z*-axis accelerometer, encoding tactile information into spiking activity that represents the intensity and timing of vibrations.

In summary, the implemented spiking neural network (SNN) utilized a layered architecture modeled with Leaky Integrate-and-Fire (LIF) neurons to replicate biologically plausible neuronal behaviors. The input layer comprised 10 nodes, including nine encoding visual color cues (conditional stimulus) and one encoding vibration cues (unconditional stimulus), they were fully connected to a second layer of 10 LIF response neurons. Each LIF neuron integrated incoming signals into its membrane potential until reaching a predefined threshold voltage (*V*_*th*_ ≈−50 mV), at which point it emitted a spike and reset its potential (*V*_*reset*_≈−65 mV). The neuron parameters included a membrane time constant (τ_*rc*_ = 20 ms) and a refractory period (τ_*ref*_ = 1 ms), chosen based on typical cortical neuron dynamics to realistically capture temporal patterns relevant for associative learning. Synaptic weights from input nodes to response neurons were initialized close to zero and adapted in real-time using Hebbian plasticity via Oja’s rule, a stabilized Hebbian learning variant. The learning rate (η = 1 × 10^−4^) was intentionally small to ensure gradual, stable weight adjustments conducive to robust associative learning. The LIF neurons projected convergently onto 9 response neurons, then aggregating their activity into one consolidated output signal. Simulations were executed with a 1 ms timestep to maintain high temporal accuracy in spike timing and neuronal dynamics. Each individual learning trial lasted 30–60 s, with experiments typically running up to 3 min in total. Throughout the trials, network inputs were dynamically updated using dedicated Nengo nodes, which continuously computed real-time red pixel ratios and vibration intensities from the robot’s camera and inertial sensor readings respectively.

At the core of the model, the associative learning layer integrates visual and vibration inputs. Each associative neuron receives input from a specific visual neuron (corresponding to a region of the visual field) and the vibration neuron. To reflect the functional roles of the Unconditioned Stimulus (US) and Conditioned Stimulus (CS), their synaptic weights are initialized to different values. The weights for the US pathway are set to a high value, typically between 0.8 and 1.0, ensuring that the vibration input alone can reliably activate the associative neurons and produce an output at the start of training. This represents the innate nature of the US, which inherently elicits a response. In contrast, the weights for the CS pathway are set to a very low value, typically between 0.01 and 0.05, ensuring that the color input alone is insufficient to activate the associative neurons at the beginning. This disparity reflects the unconditioned status of the CS at the start of training and prevents premature responses to the color input.

As training progresses, the weights for the CS pathway increase dynamically through Hebbian learning whenever the visual and vibration neurons are co-activated. The rate of this increase depends on the frequency of co-activation events and the learning rate parameter. Over time, as the CS weights grow, the color input contributes more significantly to the activation of the associative neurons. Eventually, the CS weights reach a threshold where the color input alone is sufficient to activate the associative neurons and trigger the output. This transition represents the successful acquisition of a conditioned response, where the model can respond predictively to visual cues in the absence of tactile input.

The output neuron aggregates activity from the associative neurons and produces a spiking response when the summed activity exceeds a set threshold. Initially, only the vibration input is strong enough to surpass this threshold, as the CS weights are too low to contribute significantly. However, as the CS weights grow, the color input becomes capable of independently driving the output neuron’s activity. When the output neuron spikes, it triggers the robot’s avoidance behavior, which involves moving backward in response to detecting the red wall. This behavior continues until the red color disappears from the robot’s field of vision. This dynamic output ensures that the robot adapts its behavior based on the learned association between visual and tactile inputs, allowing it to respond predictively to visual cues even in the absence of tactile input.

By carefully setting the initial weights for the US and CS pathways and adjusting them through learning, the model ensures that the associative learning process reflects both the innate significance of the US and the acquired significance of the CS. This weight dynamic, combined with the spiking-based encoding of sensory inputs, enables the model to integrate visual and tactile information effectively, supporting adaptive robotic behavior in complex environments. The use of an output neuron to control the robot’s backward movement further demonstrates the ability of this model to bridge the gap between learned associations and real-world robotic actions.

The initial weights and threshold values of the color LIF neurons were determined to balance sensitivity, stability, and learning efficiency. The high initial weights on the US pathway ensure that the vibration input reliably activates the associative neurons at the start of training, establishing the robot’s baseline reflexive response. In contrast, the low initial weights on the CS pathway prevent premature responses to visual input and focus learning on the co-occurrence of US and CS. Threshold values were tuned to ensure proper activation dynamics, with moderate thresholds for associative neurons to respond to combined inputs and higher thresholds for the output neuron to aggregate activity and trigger behavior only when necessary. These parameters are interdependent: increasing the CS weights enables visual inputs to surpass neuron thresholds after sufficient training, driving the behavioral transition from reflexive to learned responses. Together, these design choices support the gradual and biologically plausible acquisition of conditioned behavior.

The detailed structure and processes of the model are formalized in Algorithm 2. It provides a step-by-step breakdown of the initialization, input encoding, weight updates using Oja’s rule, and the behavioral transition from pre-learning (vibration-driven responses) to post-learning (color-driven avoidance). This algorithm highlights the integration of sensory inputs, the dynamics of weight adjustments, and the generation of adaptive robotic actions.

**ALGORITHM 2:** Associative Learning with Color and Vibration

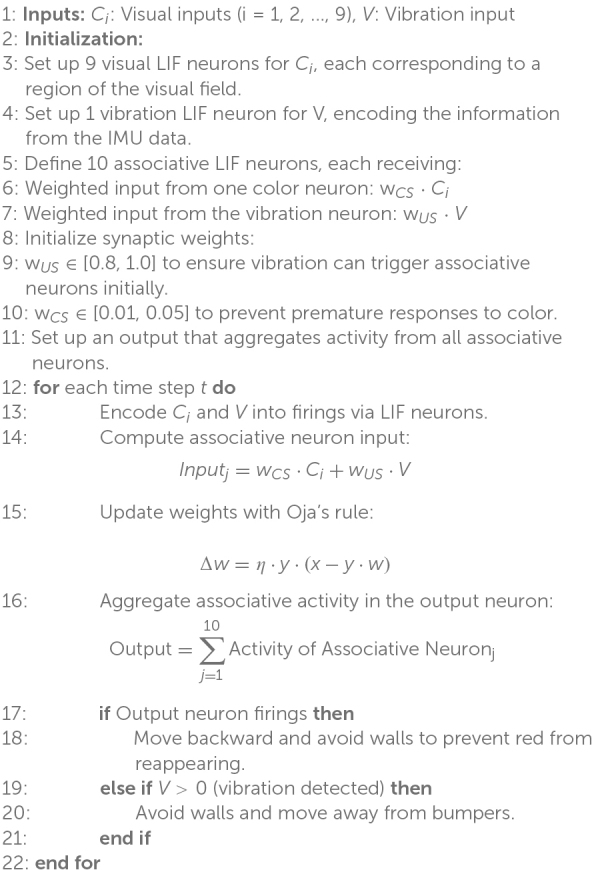

In developing the associative learning model, we studied the principles for determining synaptic weights and threshold voltages of neurons. These parameters were optimized to ensure robust learning and navigation performance while minimizing computational complexity. The synaptic weights were initialized using Hebbian principles, with the unconditional stimulus (US) pathway assigned higher weights to ensure reliable activation during initial training. The threshold voltages were tuned to balance sensitivity and stability, ensuring that neurons respond appropriately to meaningful inputs without excessive spiking. These design guidelines provide a foundation for developing more complex associative learning models in future work.

The effectiveness of the associative learning model is quantified in Algorithm 2, which charts the increase in the weight assigned to color cues over time. As the robot’s exposure to color-linked vibration areas increased, so did its reliance on visual cues for navigational decisions, demonstrating successful sensory integration and improved autonomous navigation.

The training process for the associative learning model is carefully structured to guide the system in forming a robust association between visual and tactile stimuli. The process consists of four distinct phases, designed to shape the synaptic weights between sensory and associative neurons gradually and systematically.

In the first phase, only the US, represented by the vibration signal, is presented. The vibration signal activates the vibration neuron and subsequently the associative neurons due to the high initial synaptic weights [*W*_*US*_ ∈ (0.8, 1.0)] on the US pathway. This phase establishes the robot’s baseline reflexive response, where it reacts to tactile inputs by avoiding road bumpers and walls. During this phase, the weights from the CS pathway remain unchanged, as the visual neurons are not activated.

In the second phase, only the CS, represented by the red color signal, is introduced. The visual neurons spike in response to the red signal, but the associative neurons remain inactive due to the low initial weights [*W*_*CS*_ ∈ (0.01, 0.05)] on the CS pathway. This phase ensures that the robot does not respond to visual cues alone at the beginning, confirming that the model starts from a proper baseline where the CS does not elicit a response without prior learning.

The third phase involves the simultaneous presentation of the US and CS signals, which is the critical stage for associative learning. Both the vibration neuron and the visual neurons spike together, leading to co-activation of the associative neurons. This co-activation triggers synaptic weight updates through Oja’s rule, defined as:


(14) Δ⁢W=η⋅y⋅(x-y⋅w),

where *x* represents the pre-synaptic activity from the visual or vibration neurons, *y* represents the post-synaptic activity of the associative neurons, and η is the learning rate. Over repeated training trials, the weights on the CS pathway (*W*_*CS*_) increase as the model strengthens the connections between red color signals and the associative neurons. This phase ensures that the robot begins to associate the red signal with the avoidance behavior initially triggered by the vibration signal.

In the final phase, only the CS signal is presented to test whether the learned association has been established. By this stage, the weights on the CS pathway have increased sufficiently (*W*_*CS*_ > θ_*activation*_), enabling the associative neurons to become active in response to the red signal alone. The activity of the associative neurons triggers the output neuron, leading to the robot’s avoidance behavior. The robot moves backward while avoiding walls to ensure that the red color disappears from its visual field. This phase demonstrates the successful acquisition of a conditioned response, where the robot responds to visual cues independently of tactile input.

Through these structured training phases, the model transitions from a reflexive response to tactile inputs to an adaptive response driven by learned visual cues. This process mirrors associative learning in biological systems, where repeated pairing of conditioned and unconditioned stimuli strengthens neural connections, enabling predictive responses. By employing Hebbian learning principles through Oja’s rule, the model effectively integrates visual and tactile information to support adaptive robotic behavior in complex environments.

### 3.3 Experimental validation of associative learning using neuromorphic robot

**FIGURE 14:**
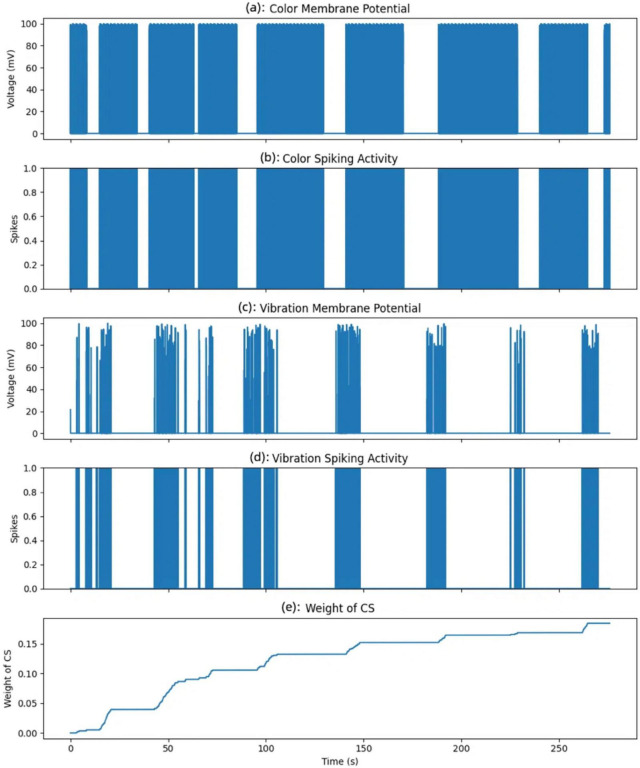
Evolution of membrane potentials, spiking activity, and conditioned stimulus (CS) weight over time. **(A)** Color membrane potential. **(B)** Color spiking activity. **(C)** Vibration membrane potential. **(D)** Vibration spiking activity. **(E)** CS weight change.

The Figure 14 tracks the associative strength between the color input from this single area and the learned response. This weight increases over time, but only when both the color and vibration neurons spike simultaneously. When the color and vibration inputs coincide and activate their respective neurons jointly, the synaptic weight associated with the color input for this neuron increases. This weight increment symbolizes the strengthening of the association between the CS and the US over time. As the weight grows, the neuron becomes more responsive to the color input alone, even without vibration, indicating a successful conditioning process.

In this experiment, the color input from one specific area (of the nine areas monitored) is treated as the CS. Initially, this color input does not elicit a response from the neuron. However, by associating it with the vibration input, which serves as the US, the model gradually learns to increase its response to the color input alone, simulating the process of associative learning. The weight change shown here represents the association strength for a single neuron corresponding to this specific color area.

The five subplots depict color membrane potential (a), color spiking activity (b), vibration membrane potential (c), vibration spiking activity (d), and weight of CS over time (e). The color membrane potential plot represents the voltage response of the neuron receiving the color input.

A spike is generated when the membrane potential exceeds the neuron’s firing threshold, shown in the color membrane potential plot. Each spike represents a moment when the color input was sufficiently strong to activate the neuron, simulating the neuron’s response to this specific area’s color input. Similarly, the vibration membrane potential and vibration spiking activity plots illustrate the response of the neuron receiving the vibration input. Unlike the color input, the vibration input displays more variability in membrane potential and more frequent spiking, reflecting the dynamic nature of the unconditioned stimulus.

**FIGURE 15:**
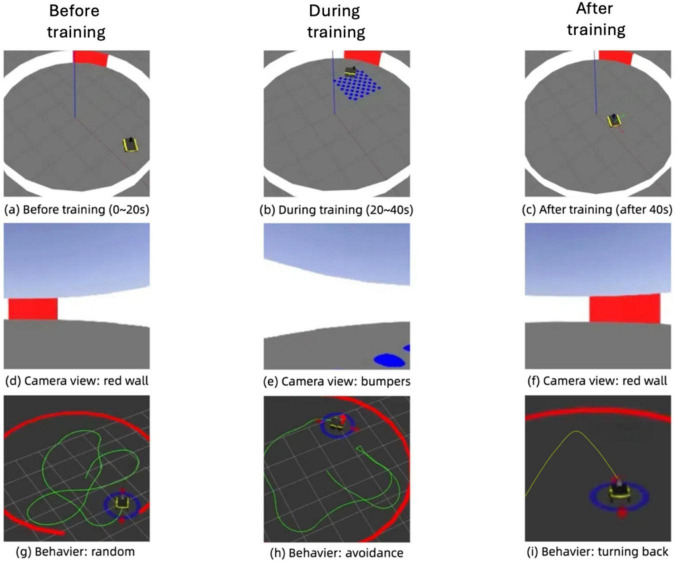
Simulation of associative learning in the robot: evolution of environment perception, camera input, and navigation behavior. **(A)** Before training (0–20 s). **(B)** During training (20–40 s). **(C)** After training (after 40 s). **(D)** Camera view: red wall. **(E)** Camera view: bumpers. **(F)** Camera view: red wall. **(G)** Behaviour: random. **(H)** Behaviour: avoidance. **(I)** Behaviour: turning back.

Figure 15 illustrates the progression of the robot’s behavior during an associative learning task in the simulation environment, showcasing its environment, camera view, and trajectory across three distinct phases: before training, during training, and after training. In the pre-training phase, the robot navigates the environment with random movement, avoiding collisions with the red wall but showing no adaptive response to the blue bumpers. Its trajectory during this stage reflects a lack of association between the visual cue (red wall) and tactile feedback (bumpers), leading to repeated interactions with the bumpers. During the training phase, the robot begins to associate the feedback from the bumpers with the red visual cue, as evidenced by hesitation near the bumpers and increased exploration in their vicinity. Post-training, the robot demonstrates a significant behavioral shift, proactively avoiding areas near the red wall. The trajectory becomes more deliberate and adaptive, steering clear of obstacles based on the learned association. The camera views across the phases reinforce this progression, capturing the robot’s evolving perception of its environment. This adaptive behavior highlights the effectiveness of integrating associative learning into the robot’s sensory processing, enabling it to navigate complex environments by using prior experiences to predict and avoid undesirable outcomes.

To rigorously evaluate our associative learning model, we performed structured real-world experiments in a controlled indoor arena. The experimental design comprised three clearly defined conditions: (1) baseline navigation (control condition), where the robot navigated an arena containing red walls and bumpers without associative learning enabled (no synaptic weight updates), establishing baseline metrics; (2) associative learning phase, where the robot repeatedly encountered simultaneous visual cues, allowing active updates of synaptic weights using Oja’s Hebbian learning rule; and (3) post-learning navigation test, where the robot navigated relying solely on previously learned visual associations without vibration cues, assessing learned avoidance behaviors.

**FIGURE 16:**
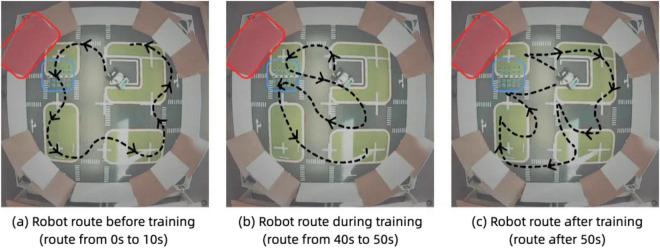
Robot trajectory with associative learning model. **(A)** Robot route before training (route from 0 to 10 s). **(B)** Robot route during training (route from 40 to 50 s). **(C)** Robot route after training (route after 50 s).

Figures 16 illustrate the robot’s navigation patterns before and after associative learning. In the pre-training phase, Figure 16A, the robot’s route is primarily determined by its ability to avoid obstacles represented by the red wall. The plotted trajectory shows that the robot successfully avoids the red wall but does not alter its behavior when encountering the blue bumpers. The robot repeatedly traverses the bumpers, as it has not yet learned to associate the red visual cue with the tactile feedback from the bumpers. This behavior reflects the unconditioned state of the model, where the robot relies solely on vibration inputs to inform its responses.

In contrast, after training Figure 16C, the robot demonstrates a learned association between the visual cue (red color) and the tactile feedback from the bumpers. The plotted trajectory shows a significant adaptation in behavior: upon detecting the red color threshold near the bumpers, the robot proactively alters its route to avoid the area altogether. This change in behavior indicates that the robot has successfully learned to associate the red color with the vibration feedback. As a result, the robot no longer needs to rely on tactile feedback alone to modify its path. Instead, it uses the visual cue as a predictive signal to avoid regions previously associated with undesirable tactile events.

**FIGURE 17:**
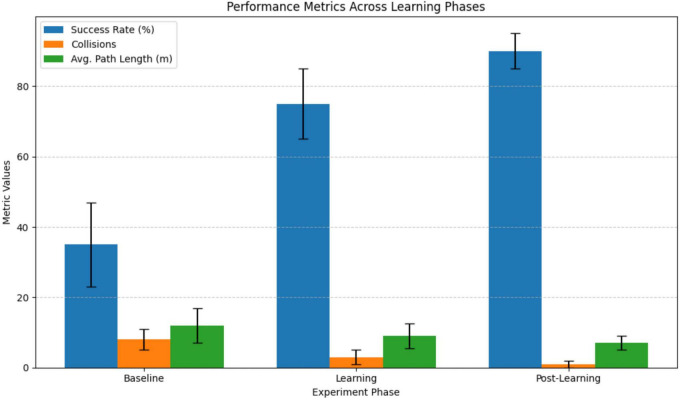
Success rate, collisions times, and path length during the experiment.

Throughout the trials, the input variables continuously recorded were visual stimuli (red pixel intensity), vibration magnitude (*z*-axis IMU acceleration), and spatial data (LiDAR-based distance mapping). Performance effectiveness was quantitatively evaluated using metrics including avoidance success rate (percentage of collision-free trials), number of passing the bumpers per trial, average path length (navigation efficiency), learning time, final average synaptic weights. Statistical analysis (paired *t*-tests) compared baseline and post-learning phases. Experimental results demonstrated clear learning effects, summarized in Figure 17 and Table 1. After learning, success rates significantly increased, collisions decreased, path efficiency improved, and stable associative learning typically occurred within approximately 10–15 exposure trials.

**TABLE 1:** Success rate, collisions times and path length during the experiment.

Phase	Success rate (%)	Collisions	Avg. path length (m)	Learning time (trials)	Synaptic weight change
Baseline	35 ± 12	8 ± 3	12.0 ± 5.0	–	–
Learning	75 ± 10	3 ± 2	9.0 ± 3.5	10–15	+0.20 (final avg. 0.70)
Post-learning	90 ± 5	1 ± 1	7.0 ± 2.0	–	–

To evaluate the robot’s learning capability, we conducted 10 real-world trials in a circular arena with road bumpers and red-colored walls placed in one quadrant. The robot’s goal was to navigate while avoiding the bumpers, using vision and vibration cues learned through associative conditioning. Learning accuracy was computed in 3-s intervals using the ratio:


(15) L⁢e⁢a⁢r⁢n⁢i⁢n⁢g⁢A⁢c⁢c⁢u⁢r⁢a⁢c⁢y=N⁢u⁢m⁢b⁢e⁢r⁢o⁢f⁢N⁢o⁢n-B⁢u⁢m⁢p⁢e⁢r⁢P⁢o⁢i⁢n⁢t⁢sT⁢o⁢t⁢a⁢l⁢P⁢o⁢i⁢n⁢t⁢s⁢i⁢n⁢ 3⁢s⁢I⁢n⁢t⁢e⁢r⁢v⁢a⁢l

**FIGURE 18:**
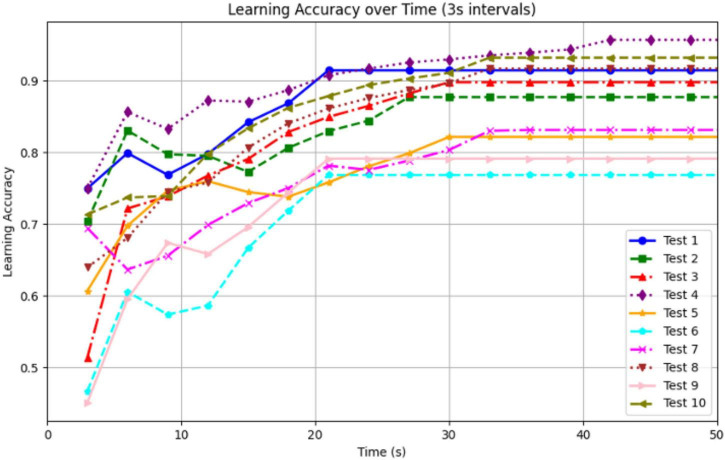
Learning accuracy over time for 10 experiments.

This metric reflects how often the robot avoided collision based on its internal learned associations, independent of direct bumper contact. As shown in the Figure 18 and Table 2, the robot exhibited a consistent and biologically plausible learning curve across all trials. Learning accuracy rose quickly in the first 10–20 s of each trial, then plateaued after approximately 30 s. Most trials reached stable performance levels between 83 and 93%, with an overall final average accuracy of 89.1% ± 3.1% (95% CI). On average, the robot required 5.6 ± 1.1 trials to achieve two consecutive bumper-free intervals. Navigation efficiency, measured as normalized path length to avoid the quadrant, improved by an average of 24.3% ± 6.4% from pre-learning to post-learning. Failure cases, defined as trials where the robot collided with bumpers after 30 s, were rare, occurring in 1 of 10 tests. This performance trend supports the effectiveness of the associative learning framework and mirrors patterns observed in animal learning tasks.

**TABLE 2:** Learning accuracy over time for 10 experiments.

Metric	Value (Mean ± SD)	95% confidence interval
Final learning accuracy (%)	89.1 ± 3.1	[86.5%, 91.7%]
Learning time (trials)	5.6 ± 1.1	[4.9, 6.3]
Navigation efficiency gain (%)	24.3 ± 6.4	[20.1%, 28.5%]
Failure rate (post-30 s collisions)	1/10 trials	–

We conducted a series of simulation and real-world experiments. These experiments were designed to replicate rodent-like associative learning in the neuromorphic robot and evaluate its performance in navigating an open-field arena. In Table 3, we provide a summary of the experimental setup, parameters, procedures, and outcomes for each experiment. This comprehensive overview ensures reproducibility and highlights the key findings of our study.

**TABLE 3:** Summary of the experiment setup and results.

Experiment	Objective	Setup	Parameters	Procedure	Outcome
Grid and place cell validation	Validate grid and place cell models.	Circular arena (radius: 1.3 m) in Gazebo simulator.	Grid cell spacing (*s_j_*): 1.0 m Orientation (θ_*j*_): π/4 Phase shifts (ϕ_*j*_): [0.5, 0] LiDAR range: 270° Linear velocity: 0.2 m/s Angular velocity: 1.5 rad/s	Robot navigated the arena while grid and place cell firing patterns were recorded.	Grid cells exhibited hexagonal firing patterns; place cells showed location-specific firing.
Associative learning in simulation	Test associative learning with visual and vibration cues.	Arena with red walls and road bumpers in Gazebo simulator.	Vibration threshold: 5 m/s^2^ Learning rate (η): 0.01 Synaptic weights: US (0.8–1.0), CS (0.01–0.05) Linear velocity: 0.2 m/s Angular velocity: 1.5 rad/s	Robot exposed to repeated pairings of red walls (CS) and road bumpers (US). After training, tested with CS alone.	Robot learned to avoid red walls without vibration signals (95% success rate).
Obstacle avoidance with associative learning	Evaluate real-world navigation with learned associations.	Physical arena (diameter: 2.6 m) with road bumpers and red walls.	Vibration threshold: 5 m/s^2^ LiDAR range: 270° Linear velocity: 0.15 m/s Angular velocity: 1.2 rad/s Learning rate (η): 0.01	Robot trained to associate red walls (CS) with road bumpers (US). After training, tested with CS alone.	Robot successfully avoided red walls in real-world tests (90% success rate).

## 4 Discussion

The adaptive behavior demonstrates the effectiveness of integrating associative learning into the robot’s sensory processing and decision-making framework. By combining visual and vibration inputs, the robot improves its ability to navigate complex environments, avoiding obstacles based on prior experiences rather than immediate feedback alone. The learned association enables the robot to respond proactively to visual cues, reducing the likelihood of collisions and improving its overall navigation efficiency.

The experiments highlight the significance of associative learning in mimicking biological spatial cognition. By employing Hebbian learning principles, the robot develops a dynamic internal representation of its environment, similar to how animals use sensory integration to navigate and adapt. Before training, the robot’s navigation was reactive, driven by immediate sensory inputs. After training, the robot exhibits predictive and adaptive behavior, demonstrating the ability to use learned associations to inform its decisions. This transition underscores the model’s capacity for real-time learning and adaptation, critical for autonomous navigation in dynamic and unpredictable environments.

In the context of our research on neuromorphic robotics, understanding how our methods align with or diverge from other neuroscience and neuromorphic engineering studies is essential. Table 4 provides a comparative overview of various studies focusing on neuronal tasks, learning methods, and validation techniques.

**TABLE 4:** Comparison with state-of-the-art associative learning work.

References	Neuron	Task	Learning method	Validation
Yang et al. (2017)	6	N/A	N/A	Simulation
Liu et al. (2016)	3	N/A	N/A	Simulation
Hu et al. (2017)	5	N/A	N/A	Simulation
Moon et al. (2014)	3	N/A	N/A	Simulation
Ziegler et al. (2012)	3	N/A	N/A	Simulation
Pershin and Di Ventra (2010)	3	N/A	N/A	Simulation
An et al. (2019)	20	N/A	Pretraining	Simulation
Zins and An (2023)	1419	Fear conditioning	No pretraining	Experiment
This work	19	Spatial learning and memory	Self-learning	Simulation & Experiment

Despite our progress, challenges remain, such as the computational demands of simulating complex neural mechanisms and the need for enhancements to perform reliably in unpredictable conditions. However, the potential benefits of this research are significant. For example, improving computational efficiency could allow real-time processing, which is essential in dynamic environments. Further integration of learning algorithms might enhance adaptability to environmental changes. Developing multi-agent systems could lead to better collaborative mapping and task execution. Adding more types of sensory inputs might create a fuller perception system. Enhancing robustness for navigation in challenging terrains could prove invaluable in areas like disaster response or planetary exploration.

The comparison in Table 5 highlights several key advantages of our associative learning model over existing neuromorphic approaches. While many models require a large number of neurons to achieve high accuracy, our approach demonstrates that efficient learning can be achieved with a minimal neural architecture. Compared to other spiking neural network-based models, which often require hundreds or even thousands of neurons, our model maintains comparable performance while significantly reducing computational complexity. For instance, the Conv-SNN model utilizes thousands of neurons, whereas our model achieves associative learning with only 19 neurons while maintaining a similar level of accuracy. This reduction in neuron count translates to lower computational and energy demands, making our approach particularly suitable for embedded neuromorphic systems and real-time robotic applications.

**TABLE 5:** Comparison with state-of-the-art Hebbian learning work.

Metric	Our work	R-STDP (Mozafari et al., 2018)	Gait imitation SNN (Ting et al., 2020)	UAV LGMD model (Salt et al., 2019)	mOSA model (Yan et al., 2021)	Conv-SNN (Abdelrahman et al., 2024)
Accuracy	90–95% (associative learning)	88.4–98.9%	N/A	80%	90%	87.5%
Convergence Speed	Seconds to minutes	N/A	10 iterations	0.1–10 ms	Seconds to minutes	0.11–0.15 s
Total neuron count	19 neurons	10 per category (80 neurons)	6 neurons (1-layer SNN)	N/A	360 neurons	6,000 neurons
Real-world experiment	Yes (tested with LIMO robot)	Yes (tested on real datasets: ETH-80)	Yes (DVS-based)	Yes (tested in UAVs)	No (computational model, no direct hardware test)	Yes (Kinova Gen3 arm)

Another advantage of our model is its ability to rapidly converge to a functional associative learning state. Unlike pre-trained models that require extensive iterations or large labeled datasets, our model adapts in real-time using biologically inspired Hebbian learning. Models based on reinforcement learning or gradient descent often require multiple training iterations before achieving stable learning outcomes. In contrast, our model reaches functional learning within seconds to minutes, which aligns with the efficiency of event-based neuromorphic models while providing a structured mechanism for integrating multiple sensory inputs. This rapid adaptation is particularly important in dynamic environments, where a robot must quickly adjust to new stimuli and update its learned associations without requiring long retraining cycles.

In addition to learning efficiency, our approach is designed for real-world deployment and has been validated in a physical robotic system. Many neuromorphic learning models remain constrained to simulations or controlled datasets, limiting their applicability to dynamic environments. By implementing our model on a neuromorphic robot in an open-field arena, we demonstrate its adaptability and robustness under real-world conditions. In contrast, some models rely exclusively on synthetic datasets such as ETH-80 or controlled environments such as UAV-based navigation, without directly interacting with complex terrain and real-world sensory variations. The integration of our model with the LIMO robot enables direct testing in an unstructured environment, where the robot successfully associates visual cues with tactile feedback and exhibits adaptive avoidance behaviors.

These factors collectively emphasize the effectiveness of our approach beyond its minimal neuron count. The ability to achieve high learning accuracy with a compact architecture, fast adaptation, and real-world validation distinguishes our model from traditional neuromorphic learning methods. The combination of associative learning with spatial memory mechanisms further enhances the robot’s ability to navigate and interact with its environment in a biologically plausible manner. The experimental validation of our model demonstrates its capability to perform robustly in real-world conditions while maintaining computational efficiency, making it a strong candidate for neuromorphic robotics applications that require self-learning capabilities in dynamic settings.

A key strength of our neuromorphic robot is its ability to adapt to new environments while retaining learned associations. For example, if the trained robot is placed in a new but similar environment—such as one with the same red color wall but a different maze shape—it will still associate the color red with an aversive stimulus and exhibit avoidance behavior. This adaptability is facilitated by the strengthened synaptic connections formed during the learning process. When the robot is returned to the initial environment, it does not require relearning, as the synaptic weights encoding the learned associations are maintained. Thus, the robot can remember multiple environments and the causal relationships between stimuli (e.g., red walls and vibrations) without additional training. This capability highlights the robustness of our associative learning model and its potential for real-world applications.

While our neuromorphic robot demonstrates promising results in replicating rodent-like associative learning, there are several limitations to our current approach. First, the scalability of our model to more complex environments with multiple sensory cues and dynamic obstacles remains to be explored. Although our model uses only 19 neurons, which reduces computational complexity, it may require further optimization to handle larger and more diverse environments. Second, the reliance on predefined synaptic weight initialization and threshold voltages may limit the adaptability of the system in scenarios where environmental conditions change rapidly. Future work could explore adaptive learning rules that dynamically adjust these parameters in real time.

Another limitation is the reliance on specific sensory inputs (vibration and visual cues) for associative learning. While these inputs are effective in controlled environments, real-world scenarios may require the integration of additional sensory modalities, such as auditory or olfactory cues, to enhance the robot’s perception and decision-making capabilities. Finally, the current implementation is limited to a single robot. Extending the model to multi-agent systems, where multiple robots collaborate and share learned associations, could open new avenues for applications in swarm robotics and distributed intelligence.

Addressing these limitations in future work will involve exploring more advanced neural architectures, integrating additional sensory modalities, and testing the system in more complex and dynamic environments. These improvements will further enhance the robustness and applicability of our neuromorphic robot in real-world scenarios.

## 5 Conclusion

This study presents a neuromorphic robot with self-learning capabilities inspired by rodent associative learning. Using Hebbian principles, the system dynamically adjusts synaptic weights in real-time, enabling self-learning and navigation. The self-learning capability of the neuromorphic robot is validated by replicating rodent-like associative learning in an open-field arena without pretraining or labeled datasets. The model is validated through simulations and real-world experiments, demonstrating the robot’s ability to adapt by associating visual cues with vibration stimuli and executing avoidance strategies. Furthermore, the neuromorphic robot integrates place and grid cell models to construct a cognitive map for navigation. The associative learning model uses just 19 neurons as perception and response units, reducing complexity and meeting SWaP constraints. Additionally, the associative model introduces principles for determining synaptic weights and neuron threshold voltages, providing design guidelines for developing more advanced associative learning models.

## Data Availability

The original contributions presented in the study are included in the article/Supplementary material, further inquiries can be directed to the corresponding author.
